# Action intersectorielle locale pendant la pandémie de COVID-19: une démarche de développement territorial en milieu rural

**DOI:** 10.17269/s41997-025-00995-w

**Published:** 2025-03-13

**Authors:** Lucie Morin, André-Anne Parent, Deena White, Christian Jetté

**Affiliations:** 1 Université de Montréal, Montréal, Québec Canada; 2 École de Travail Social, Université de Montréal, Montréal, Québec Canada; 3 Département de Sociologie, Université de Montréal, Montréal, Québec Canada

**Keywords:** Action collective, Intersectorialité, COVID-19, Théorie de l’acteur-réseau, Collective action, Cross-sector collaboration, COVID-19, Actor-network theory

## Abstract

**Objective:**

The COVID-19 pandemic affected the action of collaborative networks, connecting organizations from the public, community, and private sectors. Intersectoral action is a recognized strategy for tackling complex problems and reducing social inequalities. This study aims to understand how the COVID-19 pandemic modified local intersectoral action to improve the living conditions of rural populations.

**Methods:**

Data for this qualitative, case study were collected through semi-structured individual interviews, observation sessions, and documentary analysis. Actor-network theory was used as the theoretical framework. Data collection took place from March 2021 to June 2022. The data were processed using a thematic analysis inspired by the analytical framework.

**Results:**

The pandemic disrupted local intersectoral action, hampering networking operations and promoting a sectoral approach. Strategies favouring networking (use of technology and the liaison work of collective stakeholders) made it possible to create spaces for negotiating shared interests, identifying common causes, committing players to new roles, and sharing resources.

**Conclusion:**

When faced with disruptions, networks can be flexible, testifying to the relevance of intersectoral action to meet the needs of the population. Even if the network was in a state of near-fragmentation before the pandemic, its reconstitution and remobilization were relatively easy for the community organizers.

## Résumé

**Objectif:**

La pandémie de COVID-19 a affecté l’évolution des réseaux de collaboration plus ou moins formels, réunissant des organisations des secteurs public, communautaire et privé. L’action intersectorielle est une stratégie reconnue pour agir sur des problèmes complexes et réduire les inégalités sociales. Cette étude vise à comprendre comment la pandémie de COVID-19 a modifié l’action intersectorielle locale visant à améliorer les conditions de vie des populations rurales.

**Méthode:**

Cette étude de cas a recueilli des données à l’aide d’entretiens individuels semi-structurés, de séances d’observation et d’une analyse documentaire. La théorie de l’acteur-réseau a été utilisée comme cadre analytique. La collecte de données s’est déroulée de mars 2021 à juin 2022. Les données ont été traitées à partir d’une analyse thématique inspirée du cadre analytique.

**Résultats:**

La pandémie a affecté l’action intersectorielle locale en perturbant le fonctionnement du réseau et en valorisant l’approche sectorielle. Des stratégies favorisant la mise en réseau (utilisation de la technologie et le travail de liaison des intervenantes collectives) ont rendu possibles la création d’espaces de négociation d’intérêts partagés, l’identification de causes communes, l’engagement d’acteurs dans de nouveaux rôles et le partage des ressources.

**Conclusion:**

Lorsque confrontés à des perturbations, le réseau peut être flexible, témoignant de la pertinence de l’action intersectorielle pour répondre aux besoins de la population. Même si le réseau était dans un état de quasi-fragmentation avant la pandémie, sa reconstitution et sa remobilisation ont été relativement faciles à opérer par les intervenants collectifs.

## Introduction

Une des stratégies promues en action publique pour agir sur des problèmes complexes est l’action intersectorielle (Van Tulder & Keen, 2018). Celle-ci prend forme dans des réseaux formels et informels mobilisant des acteurs sociaux (personnes ou organisations) associés à divers secteurs d’action publique (ex.: santé, habitation, éducation) ainsi qu’à différentes sphères de la société (ex.: privée, public, communautaire et philanthropique). Les acteurs interagissent sur une base volontaire ou mandatée pour résoudre des problèmes concernant la collectivité, en accompagnant des projets dans une visée de changement social local (Kuruvilla et al., 2018). Les acteurs engagés dans des processus de collaborations intersectorielles disposent de façon asymétrique de ressources telles que des idées, du financement, de l’autorité, des expertises, et des capacités de mobilisation pour améliorer les conditions de vie de la population (Chircop et al., 2015). Selon Huxham et Vangen (2004), l’action intersectorielle génère une synergie qui produit un résultat plus substantiel que la somme des contributions des parties engagées séparément.

L’urgence sanitaire déclarée au Québec le 14 mars 2020^1^ visait à limiter la propagation de la maladie à coronavirus SARS-CoV-2 (COVID-19) pour protéger les personnes plus à risque de subir des conséquences graves s’ils contractent la maladie, c’est-à-dire les personnes âgées et celles souffrant de problèmes de santé physique ou de comorbidité (Bedford et al., 2020). Des recherches ont démontré que les personnes habitant dans des zones économiquement défavorisées avaient trois fois plus de risques d’être exposées au virus que les personnes résidant dans des zones économiquement favorisées (Lefebvre et al., 2023; Munford et al., 2022). Pour protéger la population, le gouvernement québécois et la Santé publique ont déployé diverses mesures sociosanitaires de mars 2020 à janvier 2022 comme: 1) les mesures de distanciation (le port du masque dans les endroits publics fermés et dans les services de transport collectif); 2) le confinement et les rassemblements limités; 3) le couvre-feu; 4) la fermeture des services dits non essentiels (garderies, établissements d’enseignement, commerces et industries); et 5) la vaccination (Agence de la santé publique du Canada et Réseau pancanadien de santé publique, 2020; Institut national de santé publique du Québec, 2022; Nicolas & Lepetit, 2022). L’application de ces mesures évoluait (ajout et retrait) en fonction de la propagation du virus dans les collectivités.

Malgré les contraintes associées à certaines de ces mesures nécessaires pour lutter contre la pandémie, l’action intersectorielle a été mobilisée par des regroupements d’acteurs, conceptualisés dans cet article comme des réseaux intersectoriels locaux (RIL), pour produire des actions adaptées aux réalités locales et aider les personnes en situation de vulnérabilité (Badji et al., 2023; Courtemanche et al., 2022; Lefebvre et al., 2023). L’objectif de cet article est d’examiner si la pandémie de COVID-19 a modifié l’action intersectorielle locale produite par un RIL dans un territoire rural au Québec et de comprendre le fonctionnement de ces réseaux en temps de crise.

## Méthode

### Cadre analytique

La théorie de l’acteur-réseau (TAR) est reconnue pour examiner l’agentivité d’acteurs hétérogènes (humains et non humains) dans des réseaux qui induisent des innovations pour résoudre des problèmes complexes (Latour, 2005). Selon cette théorie sociologique, l’action sociale se concrétise dans des réseaux dits «sociotechniques»—représentés par le concept de RIL dans cet article—constitués d’un ensemble d’acteurs humains (personnes et organisations) et non humains (budgets, expertises, politiques). Les RIL peuvent être plus ou moins stables et peuvent se fragmenter ou se solidifier dans le temps à travers un processus nommé «la traduction», c’est-à-dire, l’interprétation itérative et différenciée des acteurs humains et non humains qui circulent dans le RIL. Callon (1986) définit la traduction à partir de quatre processus qui peuvent se poursuivre ou se chevaucher dans les temps: la problématisation ou l’identification d’une cause commune, l’intéressement ou l’engagement des acteurs, leur enrôlement dans des rôles et responsabilités spécifiques et enfin, la mobilisation du RIL dans le temps. Ce dernier processus traduirait (ou non) un intéressement et un enrôlement réussis, où les acteurs coordonnent leurs actions permettant au RIL de se stabiliser pour un certain temps. Callon (1986) et Latour (2005) conçoivent que, pour se déplacer et évoluer, le RIL a besoin de médiateurs assumant la représentativité de celui-ci tout en veillant à ce que les rôles et les intérêts des acteurs demeurent alignés. En exerçant différents rôles, les médiateurs soutiennent la construction d’un référentiel commun et ils aident les acteurs ayant des intérêts divergents à voir la plus-value de la collaboration (Nay & Smith, 2002). Au Québec, les intervenants collectifs qui accompagnent les concertations et les initiatives sont des médiateurs. Par ailleurs, selon la TAR, le concept de controverse est fondamental. Il fait référence à la confrontation d’intérêts, de logiques et de cultures différentes, menant (ou non) à des lieux de négociation et à la conciliation par la mise en jeu de ressources et de pouvoirs d’agir asymétriques (Callon, 2006). Ce cadre d’analyse permettra d’analyser in situ les pratiques des acteurs et les interactions dans le RIL étudié afin d’expliquer les stratégies déployées permettant l’adaptation (ou non) des actions intersectorielles locales dans des conditions qui ont brusquement changé.

### Approche méthodologique et collecte de données

Les effets de la crise sociosanitaire sur l’action intersectorielle locale est l’objet d’une recherche qualitative reposant sur une étude de cas multisites (Stake, 2006) dans trois provinces canadiennes (Québec, Ontario et Alberta) et abordant des enjeux comme la réussite éducative, l’habitation et la sécurité publique. Cet article porte sur les résultats d’un projet doctoral^2^ qui représente l’un des cinq cas documentés dans la recherche pancanadienne. Il présente l’analyse d’une démarche de développement territorial visant à améliorer les conditions de vie de la population et regroupant environ 50 partenaires appartenant aux secteurs communautaire, privé et public et engagés dans différents domaines d’action publique (éducation, santé et services sociaux, emploi, habitation) dans un territoire de la région de la Capitale-Nationale au Québec. La recherche vise à expliquer l’évolution du RIL à partir du sens que les acteurs donnent à la structuration des collaborations intersectorielles pendant la pandémie de COVID-19.

Le cas étudié se situe dans un territoire rural de 4 850 km^2^ sur lequel vivent 31 011 habitants. Le RIL a pris forme en 2018 en réaction à la fin d’un financement soutenant le développement du plein potentiel des enfants de cinq ans et moins.^3^ Sur le plan du développement social, les principales organisations offrant des services sur le territoire sont: le Centre intégré universitaire de santé et de services sociaux de la Capitale-Nationale (CIUSSS-CN), une douzaine d’organismes communautaires, deux centres de la petite enfance (CPE), un office municipal d’habitation, neuf écoles primaires, une école secondaire publique et privée ainsi qu’un centre d’éducation aux adultes. Les trois principales composantes de la structure du RIL sont: 1) l’assemblée des partenaires où se décident la mission et les orientations du RIL; 2) le comité de pilotage qui coordonne le RIL; et 3) les quatre chantiers responsables d’élaborer et d’actualiser des plans d’action en habitation, mobilité, alimentation et développement de l’enfant / réussite éducative. Le programme public de lutte contre la pauvreté et l’exclusion sociale a financé l’embauche d’une chargée de projet en mobilisation citoyenne de mars 2020 à juin 2021. Le renouvellement de ce poste et l’ajout d’un poste de coordination en juin 2021 ont été financés par une organisation philanthropique. La démarche est soutenue depuis sa création par le service d’organisation communautaire du CIUSSS-CN.

La collecte de données s’est déroulée de mars 2021 à juin 2022. Les participants ont été recrutés selon la méthode d’échantillonnage raisonné (Dahl et al., 2020). Le tableau 1 détaille les trois méthodes de collecte de données utilisées (Dahl et al., 2020). La collecte et le traitement des données ont été faits par la première auteure. Trois informateurs clés ont confirmé l’exactitude des données.

**Tableau 1:** Méthodes de collecte de données

Sources	Données	Thématiques explorées
Entretiens individuels semi-dirigés N = 12 personnes travaillant dans les secteurs: communautaires, santé et services sociaux, éducation, municipal, développement économique local et habitation ainsi qu’une employée du RIL N = 3 personnes travaillant pour des organisations régionales ou provinciales qui soutiennent le RIL	Durée moyenne des entretiens: 82 min, transcris intégralement	Effets de la pandémie de COVID-19 sur les pratiques des acteurs et le fonctionnement du RIL Postures, rôles et responsabilités des acteurs
Séances d’observation 41 rencontres: Grande table Comité de pilotage Chantiers de travail Comité de coordination Comité de communication Comité de supervision	Total: 79 heures	Interactions entre les acteurs Pratiques des acteurs Ressources mobilisées
Analyse documentaire Comptes rendus des rencontres des comités énumérés dans la section «séances d’observation» Plans d’action du RIL Sondages réalisés auprès de partenaires et citoyens	Environ 50 documents	Services offerts pendant la pandémie Financement publics et philanthropiques

En utilisant le logiciel NVivo 1.7.1 pour Mac, une codification thématique (Braun & Clarke, 2006) a été effectuée en fonction des codes correspondant aux quatre processus de traduction proposés par la TAR (problématisation, intéressement, enrôlement et mobilisation) pour identifier les interactions entre les différentes entités du RIL. L’étape finale de l’analyse consistait à expliquer, dans ces termes, les changements observés dans le fonctionnement du RIL et dans les pratiques des acteurs en réaction à la pandémie de COVID-19.

### Limites

Comme toutes les entrevues ainsi que la majorité des séances d’observation ont été réalisées par visioconférence, cela représente une limite de la recherche. La collecte et le traitement des données concernant l’interaction entre les participants étaient limités par l’utilisation du logiciel Zoom.

## Résultats

Le virus et les consignes sanitaires ont affecté de différentes manières le RIL. Les résultats démontrent que les acteurs ont identifié des enjeux communs et développé des pratiques qui leur ont permis de coordonner leurs actions pendant la crise. La collaboration entre une multitude d’acteurs hétérogènes a permis de répondre aux besoins de la population alors que les institutions valorisaient davantage les actions sectorielles. Nous esquissons dans les sections suivantes les résultats de la recherche à partir des quatre processus de la traduction. Mais avant de faire cette démonstration, il est important de décrire le RIL avant le commencement de la crise sociosanitaire.

### Description du RIL avant la pandémie

Depuis mai 2019, des représentants des secteurs préscolaire et scolaire; du communautaire; de l’économie et de l’emploi; du municipal; et du développement économique local participent aux rencontres du comité de pilotage. Les six membres de ce comité se rencontrent chaque mois en présentiel pour veiller à la construction et au fonctionnement du RIL. Les orientations du RIL sont décidées lors de rencontres collectives dans lesquelles les participants sont consultés sur différents sujets. Le rôle des citoyens dans le RIL n’a pas fait l’objet de consensus entre les acteurs lors d’une de ces rencontres. La stratégie déployée par le comité de pilotage pour résoudre cette controverse a été l’embauche d’une chargée de projet en mobilisation citoyenne pour consulter les citoyens afin de connaitre leurs besoins et leurs intérêts comme l’explique une organisatrice communautaire: «*C’est un des outils qu’on a décidé d’utiliser pour de ne pas l’aborder en rencontre collective de plein front [problème de participation citoyenne]. Nous avons plutôt décidé de nous outiller avec l’embauche d’une chargée de projet*» (Q08). La chargée de projet est entrée en fonction le 19 mars 2020. Enfin, 40 acteurs du milieu prévoyaient participer à une rencontre collective le 17 mars 2020 afin de discuter des enjeux traités par le RIL.

### Problématisation: adhésion à une cause commune

Trois changements témoignent de la vision commune des acteurs par rapport aux besoins résultant de la crise sociosanitaire, témoignant d’un processus de problématisation réussi: 1) la prise en compte de la détresse psychologique dans leurs actions; 2) le soutien offert aux organismes communautaires; et 3) la création d’un bottin des services.

Dès l’annonce de la pandémie, le RIL a suspendu ses activités. Les rencontres du comité de pilotage ont repris vers la mi-avril 2020 en utilisant la visioconférence. À toutes les six semaines, les membres du comité de pilotage discutaient de leurs besoins organisationnels et des besoins de la population. La crise sociosanitaire n’a pas altéré la mission ou les orientations du RIL. Des sondages réalisés auprès des partenaires et de la population ont révélé que les besoins identifiés en octobre 2019 (sécurité alimentaire, habitation, mobilité, développement de l’enfant / réussite éducative) étaient toujours ceux qui exerçaient une grande influence sur la qualité de vie des personnes notamment celles en situation de vulnérabilité. Par exemple, des représentants des secteurs municipal et communautaire notent que les demandes d’aide alimentaire d’urgence ont «explosé». La préoccupation pour la détresse psychologique s’est ajoutée aux besoins priorisés par les acteurs. Elle découle de la menace que le virus représente pour la santé des personnes ainsi que les effets que les consignes sanitaires ont eus sur le bien-être d’une partie de la population (le stress occasionné par la conciliation famille-travail, l’isolement). Les acteurs se sont engagés dans un processus de conciliation qui s’est conclu par la définition des besoins de manière à intégrer la détresse psychologique dans les quatre enjeux déjà priorisés.

Pour répondre rapidement aux besoins exacerbés par la crise sociosanitaire, plusieurs acteurs ont soutenu trois organismes communautaires qui sont en contact avec les personnes en situation de vulnérabilité. Il s’agit des organismes communautaires offrant des services de dépannage alimentaire, de livraison de repas ou d’épicerie à domicile, d’appel d’amitié, de transport d’accompagnement et d’intervention de proximité en itinérance.

*C’étaient plus des initiatives individuelles à ce moment-là. […] Quand j’ai vu les résultats du sondage, ça m’a donné un prétexte pour appeler [la direction de] l’OMH pour lui dire: qu’est-ce que vous faites? On est là pour vous. On va débloquer des ressources pour faire des appels d’amitié à toutes les semaines pour vos locataires.* (Q08 – secteur santé et services sociaux)

Étant donné que le fonctionnement des organisations était perturbé par la présence du virus et de l’application des consignes sanitaires, certains acteurs ont voulu faciliter l’accès à l’information aux citoyens. Cette idée a plu à des représentants de la MRC et d’organismes communautaires, aux organisatrices communautaires et une bénévole qui en coordonnant leurs actions et en mettant leurs ressources (temps, argent, informations) en commun ont produit un répertoire des services qui a été distribué dans plus de 14 000 résidences dès avril 2020. Les résultats démontrent que les acteurs ont développé une vision commune des besoins et des solutions pour aider la population à traverser cette période de bouleversements.

### L’engagement: modification des intérêts et des pratiques des acteurs

La pandémie de COVID-19 a perturbé le fonctionnement du RIL d’abord par une perte de l’intérêt des acteurs pour l’action intersectorielle. La relance de l’intérêt était facilitée par deux modifications à son mode de fonctionnement: l’utilisation de la visioconférence et la modification du mandat de la chargée de projet en mobilisation citoyenne.

L’application des mesures sanitaires a provoqué un désengagement des acteurs dans le RIL dû à un surcroit de travail associé à la gestion des ressources humaines (absence maladie, conciliation travail-vie privée) et l’adaptation des services (annuler les activités de groupe, offrir des services par contact téléphonique ou par visioconférence).

*Ce n’était pas le but d’être égoïste et de travailler pour soi-même, mais ça a fermé nos œillères pour la démarche de mobilisation. Je pense qu’elle [la démarche] en a subi un coup parce qu’on n’avait pas le temps et on était en état d’urgence.* (Q07 - secteur développement économique local)

La participation des acteurs dans le RIL a été facilitée par l’introduction de nouvelles pratiques pour interagir à distance. Dès la mi-avril, la visioconférence a été utilisée pour réaliser toutes les rencontres des comités. Le RIL n’avait jamais utilisé cette technologie auparavant et les commentaires à son égard sont mitigés. La principale critique formulée à l’égard de la visioconférence est qu’elle ne permet pas les échanges informels et spontanés qui contribuent au développement des liens et de la complicité entre les acteurs comme en témoigne les propos d’un représentant du secteur de développement économique local: «*Dans les Zoom, on règle des choses pratico-pratiques, mais parfois il manque les discussions de corridor dans lesquelles on règle les vraies affaires comme on dit*» (Q11).

Le mandat de la chargée de projet en mobilisation citoyenne a été modifié en raison des consignes sanitaires dès son entrée en poste. Son rôle a été de soutenir les organismes communautaires qui offraient des services d’urgence, notamment en lien avec le dépannage alimentaire. Ce rôle imprévu a eu pour effet de stimuler les interactions entre elle et les acteurs et entre les acteurs du RIL dans l’objectif de maintenir leurs intérêts envers le RIL pendant son inactivité temporaire. Comme le mandat de l’employée a été détourné du travail de mobilisation citoyenne, les acteurs n’ont pas été en mesure de déterminer le rôle des citoyens dans le RIL dans sa première année d’existence comme l’explique une organisatrice communautaire: «*Ça a beaucoup influencé le travail en mobilisation citoyenne, mais aussi l’atteinte de nos résultats. Nous voulions sortir de ça avec carrément l’identification de mécanismes de participation citoyenne*» (Q08). La solution pour résoudre cette controverse a été de prolonger le mandat de la chargée de projet pour une autre année car la participation citoyenne est un principe fondamental pour le RIL.

### L’enrôlement: actions sectorielles et intersectorielles

Les résultats illustrent que même si l’action publique était organisée de manière sectorielle (l’information et le financement), les acteurs ont valorisé l’action intersectorielle pour augmenter leurs capacités d’agir.

Pendant la crise sociosanitaire, la circulation de l’information s’est faite davantage de manière sectorielle. C’est-à-dire que les ministères et les regroupements nationaux ou régionaux diffusaient de l’information vers les organisations locales associées à leur secteur d’activité pour soutenir l’adaptation de leurs pratiques et des services comme l’explique une représentante du secteur communautaire: «*Je fais partie aussi du regroupement des organismes communautaires familles qui nous partageait beaucoup d’informations. On pouvait s’appeler, entre nous, les directions des organismes [famille]*» (Q07).

Les financements publics ont également été administrés de manière sectorielle. Par exemple, la responsable de l’Office municipale d’habitation a bénéficié du soutien de la Société d’Habitation du Québec pour appliquer les consignes sanitaires et se procurer du matériel de protection. Un participant du secteur de l’éducation explique que l’école disposait des ressources nécessaires pour répondre à ses besoins sans dépendre de financement externe ou presque.

*[…] j’avais la marge de manœuvre pour pouvoir répondre rapidement et dire à tout mon personnel: vous avez assez des soucis avec les jeunes cassez-vous pas la tête avec l’argent. Demandez et je vais dire oui […]. Pour le bal [des finissants], on est allé chercher [de l’argent auprès] des entreprises. […]on allait chercher de l’argent pour des besoins qui ne cadrent pas dans les mesures que je peux prendre pour l’école.* (Q10 – secteur scolaire)

Confrontées à l’ampleur des besoins, certaines organisations n’étaient pas capables de répondre seules à l’ensemble des besoins qui leur étaient adressés. Les rencontres du comité de pilotage étaient le seul espace dans lequel les échanges entre acteurs de différents secteurs étaient facilités. Les acteurs ont eu recours à l’action intersectorielle afin de résoudre des problèmes complexes comme l’explique une organisatrice communautaire: «*Il y a beaucoup de choses qui sont passées par là [rencontres du comité de pilotage] et après ça par l’informel quand on se dit «OK, on prend rendez-vous pour en reparler ensemble»* (Q08).

Une autre stratégie pour renforcer l’action intersectorielle pendant la pandémie a été l’accompagnement des intervenantes collectives. Une organisatrice communautaire a mentionné: «*On a contacté tous ces organismes et nous avons dit qu’on était les personnes pivots à contacter s’il y avait des problèmes COVID»* (Q09). Les organisatrices communautaires ont: 1) relayé les informations concernant les consignes sanitaires et les occasions de financement aux organismes communautaires; 2) fait des états de situation aux équipes du CIUSSS-CN et à l’équipe de santé publique sur les besoins de la population et des organismes communautaires; et 3) effectué des approches personnalisées auprès des acteurs pour connaitre leurs besoins tout en les encourageant à solliciter d’autres organisations et réseaux pouvant leur apporter du soutien. Le travail de liaison des organisatrices communautaires et de la chargée de projet ainsi que les rencontres du comité de pilotage ont contribué aux interactions entre une diversité d’acteurs, d’organisations et de réseaux dans un contexte caractérisé par la fragmentation de l’action par secteur.

### Solidification des liens

Même si la pandémie de COVID-19 a entravé la réalisation de certaines actions, notamment par la perte d’intérêt des acteurs dans un premier temps pour l’action intersectorielle, le contexte de crise a contribué finalement à la reconnaissance de la pertinence de l’action intersectorielle locale.

L’application des mesures sanitaires a empêché le déploiement de certaines actions planifiées par le RIL comme la visite d’une école primaire aux enfants fréquentant un CPE pour faciliter leur transition entre les deux établissements.

La crise sociosanitaire a généré des interactions spontanées entre des acteurs à l’intérieur et à l’extérieur du RIL comme en témoignent les propos d’une représentante du secteur communautaire: «*Je recevais des courriels de personnes qui ne m’ont jamais parlé pour me dire: tel fonds, tel fonds, tel fonds est disponible. Cela a donné lieu à beaucoup d’entrées d’argent […]»* (Q07) et d’une représentante du secteur de l’habitation: «*Un concessionnaire [automobile] nous a offert des masques pour les aînés […]. Il y a eu beaucoup de bénévoles qui ont fait du transport pour mes locataires pour qu’ils puissent se faire vacciner […]»* (Q12). La multiplication des interactions entre les acteurs et les organisations du milieu contribuent à renforcer le RIL.

Les participants croient que le RIL peut contribuer à la résilience du milieu lors des prochaines crises comme le souligne une participante:

*Si la démarche avait été un peu plus avancée, d’admettons six mois ou un an, au moment de la pandémie ça aurait été merveilleux parce que ce réseautage-là aurait déjà été là. On aurait eu nos chantiers et ça aurait probablement accéléré des choses ou permis d’avoir des solutions auxquelles on ne pouvait pas penser parce qu’on n’était pas réseauté.* (Q09 – secteur de la santé et des services sociaux)

## Discussion

Cette section vise à répondre à la question de recherche en expliquant les effets de la crise sociosanitaire sur l’action intersectorielle locale et en identifiant les stratégies mobilisées par les acteurs pour assurer le fonctionnement du RIL.

### Effet de la pandémie sur l’action intersectorielle

La crise sociosanitaire a affecté le fonctionnement du RIL en entravant la réalisation d’actions planifiées avant le déclenchement de la pandémie (rencontre collective planifiée le 17 mars pour discuter des enjeux et l’activité de transition du CPE vers l’école) et en forçant la modification du mandat de la chargée de projet en mobilisation citoyenne. Le report de ces activités a affecté différemment le processus de consolidation du RIL. Bien que la consultation des partenaires ait été faite par des sondages en ligne, le processus décisionnel quant au rôle des citoyens dans le RIL n’a pas été complété par la chargée de projet dans son premier mandat. Pendant plusieurs mois, la voix des citoyens n’a pas été entendue; des idées et des savoirs n’ont pas été intégrés dans l’analyse des besoins privant ainsi le RIL de ressources précieuses pour adapter ses actions à ce contexte extraordinaire (Dewulf & Elbers, 2018).

En plus d’altérer le fonctionnement du RIL, la pandémie de COVID-19 a ralenti son processus de construction. Le processus de traduction de la TAR a mis en lumière que pour demeurer opérationnels pendant la crise, les acteurs avaient besoin de connaitre les consignes sanitaires à respecter, qui variaient en fonction du taux de personnes contaminées par le virus, et de mobiliser des ressources comme de l’argent, du matériel de protection, et des savoirs. L’accès à ces ressources s’est fait selon des approches sectorielles dans lesquelles les ministères et les regroupements nationaux ou régionaux transmettaient des ressources aux organisations locales de leur secteur d’activité (habitation, éducation). Le fonctionnement structurel de l’action publique a favorisé l’approche sectorielle dans les collaborations, provoquant ainsi une perte d’intérêt temporaire des acteurs pour l’action intersectorielle locale. Les travaux de Shareck et al. (2024) soulignent également que le contexte de crise a provoqué la fermeture de certains acteurs à l’action intersectorielle.

Dans un contexte de sectorisation de l’action publique, ce n’est pas le RIL, mais bien les organismes communautaires qui disposaient de la crédibilité et de la légitimité pour répondre aux besoins. Des entreprises privées, des organisations publiques et des citoyens ont offert aux organismes communautaires diverses ressources (argent, matériel de protection, temps) pour les aider à répondre aux besoins urgents de la population. En raison de leur proximité relationnelle avec les personnes en situation de vulnérabilité et les autres organisations du territoire ainsi que leur rapidité d’exécution, les organismes communautaires bénéficiaient d’une grande capacité d’action (den Broeder et al., 2022; Ndumbe-Eyoh et al., 2021; Shareck et al., 2024). Paradoxalement, la reconnaissance octroyée aux organismes communautaires a eu pour effet d’affaiblir l’action intersectorielle locale au début de la crise. Ce sont les productions et les représentations faites par un RIL qui lui permettent de bâtir sa légitimité et sa crédibilité auprès des réseaux afin d’obtenir les appuis et les ressources dont il a besoin (Bilodeau et al., 2022). On constate que la pandémie a ralenti le processus de construction du RIL en détournant momentanément l’intérêt des acteurs pour l’action intersectorielle locale. Cependant, la réponse aux besoins pendant la crise a présenté un niveau de complexité qui a mis en évidence les limites de l’action sectorielle.

### Stratégies en faveur de l’action intersectorielle

Les résultats démontrent que les acteurs ont rapidement adhéré à une vision commune des besoins découlant de ce contexte singulier. Leur désir de répondre aux besoins des personnes en situation de vulnérabilité a facilité les processus de négociation d’intérêts et de responsabilités qui se sont traduits par l’intégration transversale de la détresse psychologique dans les enjeux abordés dans les quatre chantiers du RIL ainsi que par la création et la diffusion d’un répertoire des services.

Les mesures sanitaires ont forcé les acteurs à modifier leurs pratiques pour évoluer dans cette nouvelle réalité. Les acteurs ont utilisé les rencontres du comité de pilotage comme espace pour discuter de leurs besoins organisationnels et des besoins de la population. Cette nouvelle pratique a permis d’améliorer la communication entre les acteurs. Comme le mentionnent Stephen et al. (2023), le partage d’informations sur leurs organisations respectives et la mise en réseau des acteurs contribuent à la création et à la bonification d’idées qui sont à l’origine d’innovations permettant de résoudre des problèmes complexes. En adoptant cette nouvelle pratique, les acteurs ont voulu que les bonnes choses se produisent au bon moment et de manière durable en tentant d’aligner les structures, les processus, les résultats et les responsabilités pour que l’action intersectorielle locale offre une réponse adaptée aux besoins de la population (Huxham & Vangen, 2005).

Les interactions entre les membres du RIL se sont faites, pendant plusieurs semaines, uniquement de manière virtuelle (visioconférence, sondage en ligne). Bien que l’utilisation de technologies de communication ait permis aux acteurs de reprendre contact, celles-ci n’offrent pas les conditions nécessaires à la consolidation de l’action intersectorielle locale. Selon Skopp et al. (2015), la rencontre en présentiel est considérée comme le moyen de communication le plus complet, car en plus de permettre une rétroaction immédiate sur l’information partagée, elle favorise la cohésion, la confiance et les interactions entre les acteurs. Les travaux de Jochems (2022) ainsi que Deshaies et Cloutier (2022) ont démontré que le recours à la visioconférence pendant la pandémie est venu modifier les communications entre les acteurs provoquant une diminution significative des relations informelles entre ceux-ci en plus de provoquer une perte du sens de leurs pratiques professionnelles et de diminuer leurs capacités à mobiliser des acteurs dans des actions concertées. Le recours à la technologie a contribué au maintien de l’engagement des acteurs dans le RIL par le partage de l’information, mais il a toutefois fragilisé l’action intersectorielle locale en ne permettant peu ou pas l’augmentation de la confiance et de la cohésion entre les acteurs qui se développe davantage dans des contacts informels et spontanés (Shareck et al., 2024).

L’adhésion forte des acteurs à une cause commune, des interactions continues et la mise au jeu des ressources se sont traduites dans la mise en œuvre de solutions collectives et dans la résolution des tensions qui auraient pu entraver l’action intersectorielle locale (Bilodeau et al., 2022). Les stratégies de mise en réseau des acteurs se sont avérées efficaces et innovantes.

## Conclusion

Cet article vise à démontrer les effets que la COVID-19 a eus sur l’action intersectorielle locale dans un RIL d’une communauté rurale au Québec, à l’aide du déploiement de la TAR. Les résultats démontrent que le virus et l’application des consignes sanitaires ont perturbé le fonctionnement du RIL mais, à travers des processus de traduction, les acteurs étaient en mesure d’adopter de nouvelles pratiques et de renforcer l’action intersectorielle. La construction du RIL a été ralentie par la privation de ressources essentielles à sa consolidation. Malgré cela, la crise sociosanitaire a provoqué une forte mobilisation des acteurs pour une cause commune et a contribué à amplifier les interactions entre les acteurs dans la collectivité. Les stratégies de mise en réseau (flexibilité dans le mode de fonctionnement et utilisation de la technologie) mobilisées par les acteurs ont soutenu l’action intersectorielle locale en créant des espaces de négociation d’intérêts partagés, d’identification de causes communes, d’engagement d’acteurs dans de nouveaux rôles et à la mise au jeu de ressources.

Même lorsqu’ils sont confrontés à des conditions contraignantes, les RIL peuvent faire preuve d’une flexibilité élevée témoignant de la pertinence de l’action intersectorielle pour répondre aux besoins de la population. Même si le RIL était dans un état de quasi-fragmentation avant la pandémie, sa reconstitution et sa remobilisation ont été relativement faciles à opérer par les intervenantes collectives. La pandémie de COVID-19 était caractérisée par une urgence d’agir. Il serait intéressant de documenter comment les RIL peuvent rapidement et pertinemment problématiser des enjeux urgents comme la lutte contre les changements climatiques sans être pressés par l’ajout ou le retrait rapide d’acteurs non humains provoqués par la situation de crise. Comment les intervenants collectifs peuvent-ils réengager les acteurs dans de nouveaux rôles pour agir sur un enjeu certes urgent, mais jugé non prioritaire par ces mêmes acteurs qui peinent à répondre aux besoins de base de la population? L’exploration de ces questions de recherche est essentielle pour supporter les acteurs dans leur lutte contre les inégalités sociales, environnementales et climatiques qui affligent les personnes en situation de vulnérabilité.

## Contributions à la connaissance

Qu’est-ce que cette étude ajoute aux connaissances existantes?

- Même si l’action intersectorielle est une stratégie reconnue pour réduire les inégalités sociales de santé et améliorer les conditions et la qualité de vie des personnes, elle se trouve freinée par certaines politiques publiques qui organisent l’action sociale selon une approche sectorielle.
- Comme la réponse à certains besoins complexes exige l’action intersectorielle, des acteurs proactifs ont eu recours à des stratégies de mise en réseau pour renforcer les dynamiques de collaboration intersectorielle.

Quelles sont les principales conséquences qu’il y a lieu d’en tirer pour les interventions, la pratique ou les politiques en santé publique?

- Les acteurs auraient avantage à développer et à consolider des mécanismes de circulation d’information, de coordination de l’action et de financement en réseau pour compléter leurs structures sectorielles existantes afin d’avoir des collectivités plus résilientes en contexte de crise.
- Les politiques et programmes publics devraient appuyer l’action intersectorielle locale en finançant l’accompagnement qui permet la création et de consolidation de RIL engagés dans les luttes contre les crises climatiques, écologiques et sociales.

## Footnotes

1 Source: https://www.quebec.ca/nouvelles/actualites/details/pandemie-de-covid-19-le-gouvernement-du-quebec-declare-letat-durgence-sanitaire-interdit-les-visites-dans-les-centres-hospitaliers-et-les-chsld-et-prend-des-mesures-speciales-pour-offrir-des-services-de-sante-a-distance

2 Numéro d’approbation éthique du comité d’éthique de la recherche Société et culture de l’Université de Montréal: CERSC-2021–018-D.

3 Source: https://fondationchagnon.org/initiatives-soutenues/territoires/avenir-denfants/

## Déclarations

### Financement

L’autrice principale a reçu une bourse de recherche doctorale du Fonds de Recherche du Québec—Société et culture (FRQSC).

### Conflit d’intérêts

Aucun conflit d’intérêts à déclarer.

### Approbation éthique

Les procédés appliqués dans les études impliquant des sujets humains étaient conformes aux normes éthiques du comité de la recherche interne et/ou national et à la Déclaration d’Helsinki de 1964 et à ses modifications ultérieures ou à des normes éthiques comparables. Numéro d’approbation éthique du comité d’éthique de la recherche Société et culture de l’Université de Montréal: CERSC-2021–018-D.

### Consentement à participer

Nous avons obtenu le consentement éclairé de toutes les personnes ayant participé à l’étude.

### Consentement pour la publication

Non applicable.

### Disponibilité des données et du matériel

Non applicable.
